# Metabolomics-assisted evaluation of differences and clinical value between ablation and surgical treatment for lung adenocarcinoma

**DOI:** 10.3389/fonc.2026.1836590

**Published:** 2026-07-16

**Authors:** Youli Wen, Yang Zeng, Wenqiang Li, Xinyu Deng, Wu Qunhua, Zhiping Deng

**Affiliations:** Department of Respiratory and Critical Care Medicine, Zigong First People’s Hospital, Zigong, China

**Keywords:** ablation, lung adenocarcinoma, MALDI-MS, metabolomics, surgery

## Abstract

The optimal transition from surgery to minimally invasive ablation for early lung adenocarcinoma requires elucidation of their differential metabolic impacts and clinical benefits. Using MALDI-MS platform, this study investigated 40 patients (15 ablation, 25 surgery) to characterize treatment-specific metabolic signatures and evaluate diagnostic performance. Following rigorous matrix validation, metabolomic profiling revealed distinct metabolic networks, with supervised OPLS-DA models achieving effective group separation and identifying numerous differentially expressed metabolites. For treatment discrimination, metabolomics demonstrated superior diagnostic efficacy (AUC = 0.79) compared to traditional imaging (0.75) and biochemical tests (0.65). Notably, integrating all three modalities substantially enhanced discriminatory power (AUC = 0.85), highlighting the value of multi-dimensional information fusion. Clinical assessment under balanced baseline conditions showed significant advantages for ablation: shorter hospital stays (P = 0.003), reduced costs, and fewer complications, while maintaining equivalent pulmonary function (FEV1/FVC). These findings provide integrated metabolomic and clinical evidence supporting ablation as a minimally invasive, efficacious alternative to surgery, offering molecular insights into treatment-induced metabolic reprogramming and establishing a foundation for optimized therapeutic decision-making in early lung adenocarcinoma.

## 1 Introduction

As a malignant tumor with high incidence worldwide, the prevention, control, diagnosis, and treatment of lung cancer have always been key focuses in the medical field (1–3). Data from the Global Cancer Report released in 2021 indicates that lung cancer incidence remains the highest among global malignant tumors, with its mortality rate further climbing to 21.5%. Moreover, in 93 countries and regions globally, lung cancer is the leading cause of cancer death among men, posing a serious threat to human life and health. Currently, although some progress has been made in the clinical diagnosis and treatment of lung cancer, issues such as insufficient sensitivity in early diagnosis and the lack of quantitative evaluation for the advantages and disadvantages of different treatment options continue to hinder the improvement of patient prognosis (4–6). Exploring efficient diagnostic methods and establishing a scientific treatment evaluation system have become core research directions in the field of lung cancer diagnosis and therapy.

In recent years, metabolomics technology has developed rapidly and a growing body of scientific research confirms that metabolite combinations can serve as novel biomarkers, providing reliable evidence for the early screening and precise diagnosis of lung cancer, effectively compensating for the shortcomings of traditional diagnostic methods, and offering new insights and perspectives for malignant tumor diagnosis and treatment (7–9). Along with these advances, substantial breakthroughs have been achieved in metabolomics-based lung cancer research. For instance, novel metabolite signatures have been successfully used to distinguish lung adenocarcinoma from healthy controls and to predict responses to targeted therapy and immunotherapy (10–12). However, a critical research gap remains: the metabolic differences between local thermal ablation and conventional surgery for early-stage lung cancer have not been systematically compared, and no metabolite panel has been established to evaluate the clinical advantages of ablation over surgery.

Among these, Matrix-Assisted Laser Desorption/Ionization Mass Spectrometry (MALDI-MS), as a core technology in metabolomics research, has become the preferred method for clinical tumor metabolite detection due to its advantages such as fast detection speed, simple sample preparation, high sensitivity, strong salt tolerance and anti-interference capability (13–15), and suitability for high-throughput analysis. It is well-suited for the rapid analysis of complex clinical biological samples, enabling the precise capture of subtle metabolic changes in the body, and providing efficient technical support for screening metabolism-related biomarkers and evaluating therapeutic efficacy in lung cancer. Regarding treatment, surgery is the classic approach for early-stage lung cancer. Particularly, the widespread adoption of video-assisted thoracoscopic surgery (VATS) has significantly optimized treatment outcomes, showing positive effects in reducing postoperative complication rates, improving patients’ quality of life, and lowering mortality (16–18). However, surgery still has limitations, including significant trauma, prolonged postoperative recovery, and a restricted applicable population, creating an urgent clinical need for more minimally invasive and efficient treatment options.

Local thermal ablation, a precise minimally invasive technique, has been gradually applied in the clinical treatment of early-stage lung cancer due to multiple advantages such as minimal trauma, definite efficacy, high safety, strong repeatability, and a wide range of applicable patients. It encompasses mainstream modalities like radiofrequency ablation (RFA), microwave ablation (MWA), and cryoablation, with the number of clinical applications increasing rapidly year by year (19, 20). However, currently, there is a lack of comparative clinical studies on thermal ablation versus surgery for treating pulmonary nodules. Key questions regarding differences in metabolic characteristics and comparisons of clinical benefits between the two procedures remain unclear.

**Scheme 1:**
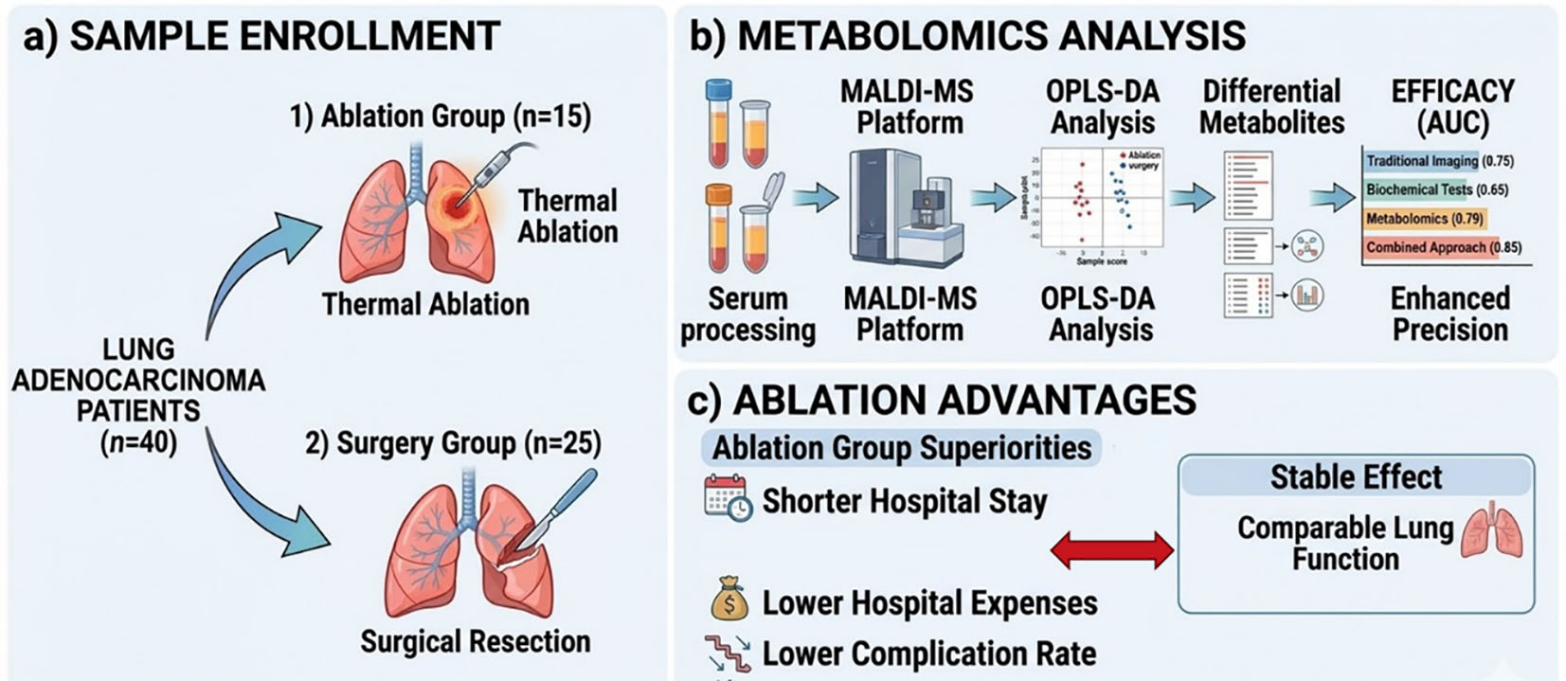
Schematic illustration of this study. **(a)** A total of 40 patients with lung adenocarcinoma were enrolled and divided into an ablation group (n=15) and a surgery group (n=25), with balanced baseline characteristics. **(b)** Metabolomic analysis was performed using mass spectrometry technology. Multivariate statistical models were employed to compare metabolic profiles between the two groups and to evaluate diagnostic performance alongside traditional imaging and biochemical tests. **(c)** The results revealed significantly different metabolic signatures between the ablation and surgery groups. Combining metabolomics with traditional methods improved the diagnostic AUC for treatment differentiation to 0.85. Clinically, the ablation group demonstrated shorter hospital stays, lower costs, and fewer complications, while achieving pulmonary function preservation comparable to surgery, highlighting its minimally invasive advantages and clinical value

Therefore, we collected clinical samples (15 ablation vs. 25 surgery; **Scheme 1a**) for metabolomic profiling (**Scheme 1b**) to ultimately assess the clinical advantages of ablation therapy (**Scheme 1c**). In this study, relying on mass spectrometry technology combined with metabolomic analysis, aims to deeply investigate the metabolic differences in lung adenocarcinoma patients following ablation therapy versus surgical treatment. Simultaneously, clinical indicators of the two procedures will be compared, and the diagnostic value of metabolomics combined with traditional imaging and biochemical tests will be evaluated. The goal is to provide a scientific basis and practical reference for the precise diagnosis and treatment of pulmonary nodules and the optimization of treatment strategies.

## 2 Materials and methods

### 2.1 Study subjects and sample data

This study was approved by the hospital’s Medical Ethics Committee, and all participants provided informed consent by signing the consent form. Inclusion criteria: Pathologically confirmed lung adenocarcinoma, meeting indications for surgery or thermal ablation; complete clinical data; good compliance, able to cooperate with subsequent testing and follow-up. Exclusion criteria: Presence of other malignant tumors; severe dysfunction of vital organs such as the heart, liver, or kidneys; receipt of anti-tumor interventions like radiotherapy, chemotherapy, or targeted therapy before the procedure; unqualified sample collection or missing data.

A total of 40 patients with lung adenocarcinoma were enrolled and divided into two groups according to the treatment method: an ablation group (15 cases) and a surgery group (25 cases). Clinical baseline data (age, sex, disease stage, etc.), treatment-related samples, and clinical outcome data such as hospital stay, hospitalization costs, complication rates, and pulmonary function indicator (FEV1/FVC) were collected for both groups (3, 21–23). Sample collection, storage, and pretreatment procedures were standardized to ensure the accuracy and reliability of subsequent experimental data.

### 2.2 MALDI-MS analysis of standard analytics under salt and protein interference

To validate the detection performance of the matrix, phenylalanine and glucose standards were analyzed. The mass spectra exhibited clear sodium ([M+Na]^+^) and potassium ([M+K]^+^) adduct peaks, confirming the detection sensitivity of the matrix for small molecules. For quantitative assessment, glucose standards at concentrations ranging from 0.1 to 0.8 µM were prepared. To evaluate the anti-interference capability of the matrix, mass spectra were acquired from samples containing high-concentration salt ions (8 mM NaCl) and protein impurities (0.5 mg/mL BSA) (8). The obtained mass spectra of target analytes in the presence of 8 mM NaCl and 0.5 mg/mL BSA showed no significant signal attenuation or peak distortion, confirming the matrix’s robust anti-interference capability.

### 2.3 Matrix performance validation for mass spectrometry

CHCA (α-cyano-4-hydroxycinnamic acid) was selected as the matrix. (7, 24) Phenylalanine and glucose were used as standards. Serial concentrations of standard solutions were prepared and detected using the established matrix by mass spectrometry. The appearance of sodium and potassium adduct peaks for the standards was observed to evaluate detection sensitivity. Glucose solutions with a concentration gradient of 0.1–0.8 μM were prepared for linearity testing to validate the quantitative detection capability of the matrix. Furthermore, to simulate a complex detection environment, salt ions and protein impurities were added, and the changes in the mass spectrometry signals of the target analytes were monitored to assess the matrix’s tolerance to salt and protein interference.

### 2.4 Metabolomic analysis and data processing

Metabolites were extracted from patient samples in both groups and detected by mass spectrometry. Mass spectrometry data were preprocessed using XCMS software with the following parameters: peak width = 5–20 s, signal-to-noise ratio threshold = 6, and retention time correction using obiwarp algorithm. Data were then normalized by total ion current and log-transformed prior to statistical analysis. Multidimensional statistical analysis was performed using principal component analysis (PCA), t-SNE analysis, and OPLS-DA models to explore differences in metabolic characteristics between the two groups (25–30). PCA is an unsupervised method that reduces dimensionality by maximizing variance without using class labels, making it suitable for exploratory analysis and outlier detection; in contrast, both PLS-DA and OPLS-DA are supervised approaches that incorporate class information to maximize separation between groups. PLS-DA is effective for moderate group differences, while OPLS-DA further separates class-orthogonal variation to enhance discrimination power when within-group variability is high; all three methods require data centering and scaling, and the supervised models demand rigorous cross-validation to avoid overfitting. For OPLS-DA model validation, 7-fold cross-validation and 200 permutation tests were applied; variables with variable importance in projection (VIP) > 1.0 and |log2 fold change| > 1.0 were considered as potential differential metabolites. Differentially expressed metabolites were identified using heatmaps and volcano plots to clarify the pattern of metabolic profile differences. Concurrently, data from traditional imaging and biochemical tests were collected. The area under the curve (AUC) was calculated for each individual method and for their combination to evaluate the diagnostic efficacy for differentiating the treatment types. AUC confidence intervals were estimated using 1,000 bootstrap resamples with the DeLong method, and the optimal cutoff was determined by maximizing the Youden index. (31–34).

### 2.5 Statistical analysis of clinical indicators

SPSS statistical software was used for data processing. Measurement data were expressed as mean ± standard deviation, and comparisons between groups were performed using the t-test. Count data were expressed as rates (%), and comparisons between groups were performed using the χ² test. P < 0.05 was considered statistically significant (1). Indicators such as age, hospital stay, hospitalization costs, complication rates, and FEV1/FVC were compared between the two groups to assess differences in clinical benefits between the two procedures.

## 3 Results

### 3.1 Performance validation of the mass spectrometry matrix

**Figure 1:**
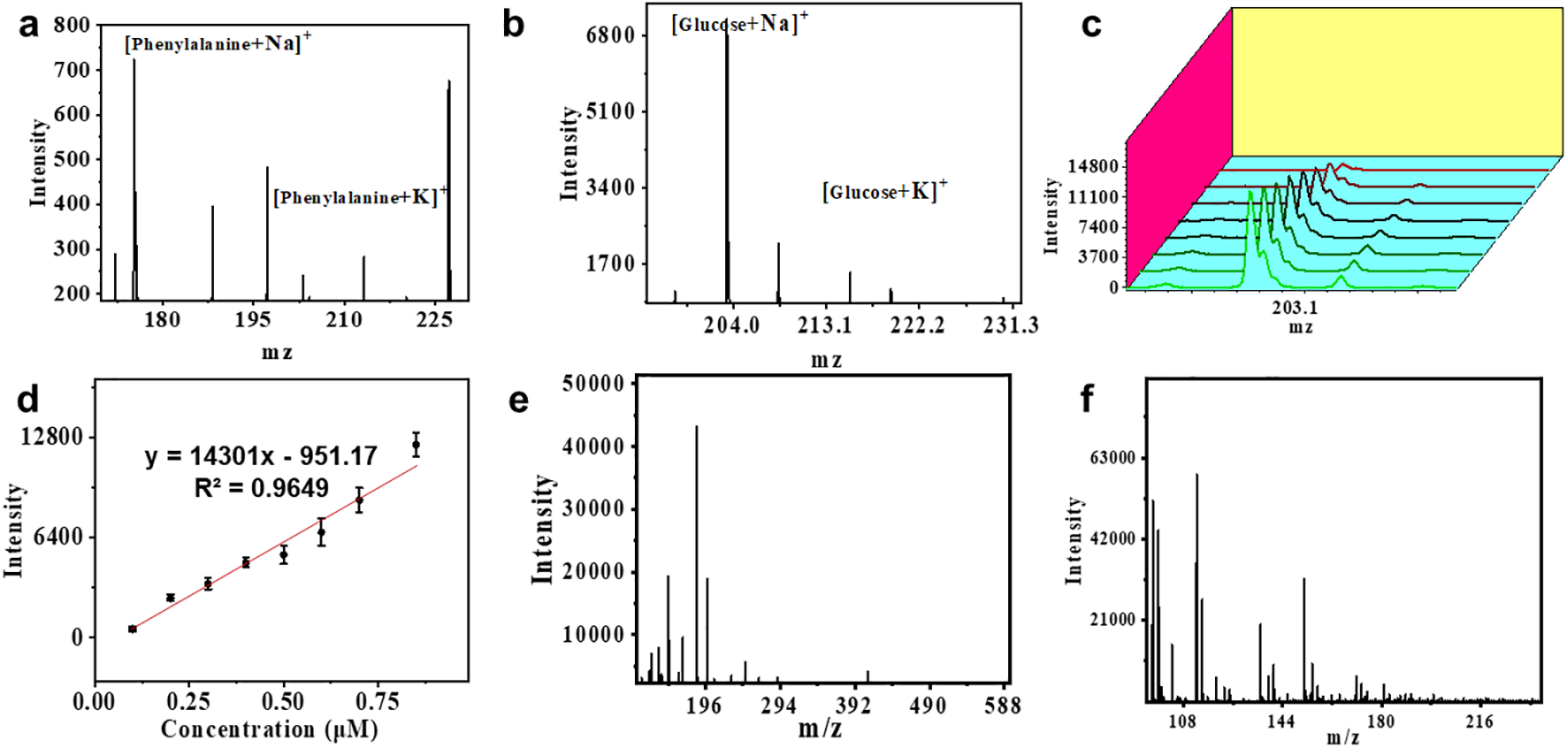
Performance validation of the MALDI-MS matrix. **(A, B)** Mass spectra showing clear sodium and potassium adduct peaks for phenylalanine and glucose standards, confirming detection sensitivity. **(C, D)** Linear regression analysis for glucose detection within the 0.1–0.8 μM concentration range, demonstrating favorable linearity (R² > 0.99) suitable for quantification. **(E, F)** Mass spectra of target analytes in the presence of salt ions (8 mM NaCl) and protein impurities (0.5 mg/mL BSA), showing no significant signal attenuation or peak distortion, confirming the matrix’s robust anti-interference capability.

In MALDI-MS analysis, the matrix absorbs ultraviolet laser energy and facilitates the desorption and ionization of analytes by transferring protons or charges, which is essential for detecting small molecules that otherwise ionize poorly. Phenylalanine and glucose were selected as standards because they are readily available, easy to standardize, and commonly used to evaluate MALDI-MS performance based on sodium ([M+Na]^+^) and potassium ([M+K]^+^) adduct formation. To verify the importance of the mass spectrometry matrix for metabolite detection, this study systematically evaluated the matrix using phenylalanine and glucose as standards. The detection results showed clear and significant sodium and potassium adduct peaks for both standards, with regular peak shapes and no significant interference from impurity peaks, as shown in **Figures 1A, B**. This fully demonstrates that the matrix possesses good performance for metabolite detection, enabling accurate identification of target metabolite signals.

For quantitative detection, glucose solutions in the concentration range of 0.1-0.8 μM were tested. The results confirmed a favorable linear relationship and good fit for glucose detection signals within this concentration range, as shown in **Figures 1C, D**, meeting the requirements for quantitative metabolite analysis. Results from salt tolerance and protein interference experiments indicated that in complex environments containing salt ions and protein impurities, the mass spectrometry signal intensity of the target analytes showed no significant attenuation, and the peak shapes were not substantially disturbed, as shown in **Figures 1E, F**. This further confirms the excellent anti-interference ability of the matrix, making it suitable for detecting complex clinical samples.

### 3.2 Metabolomic differences in lung adenocarcinoma patients after ablation and surgery

**Figure 2:**
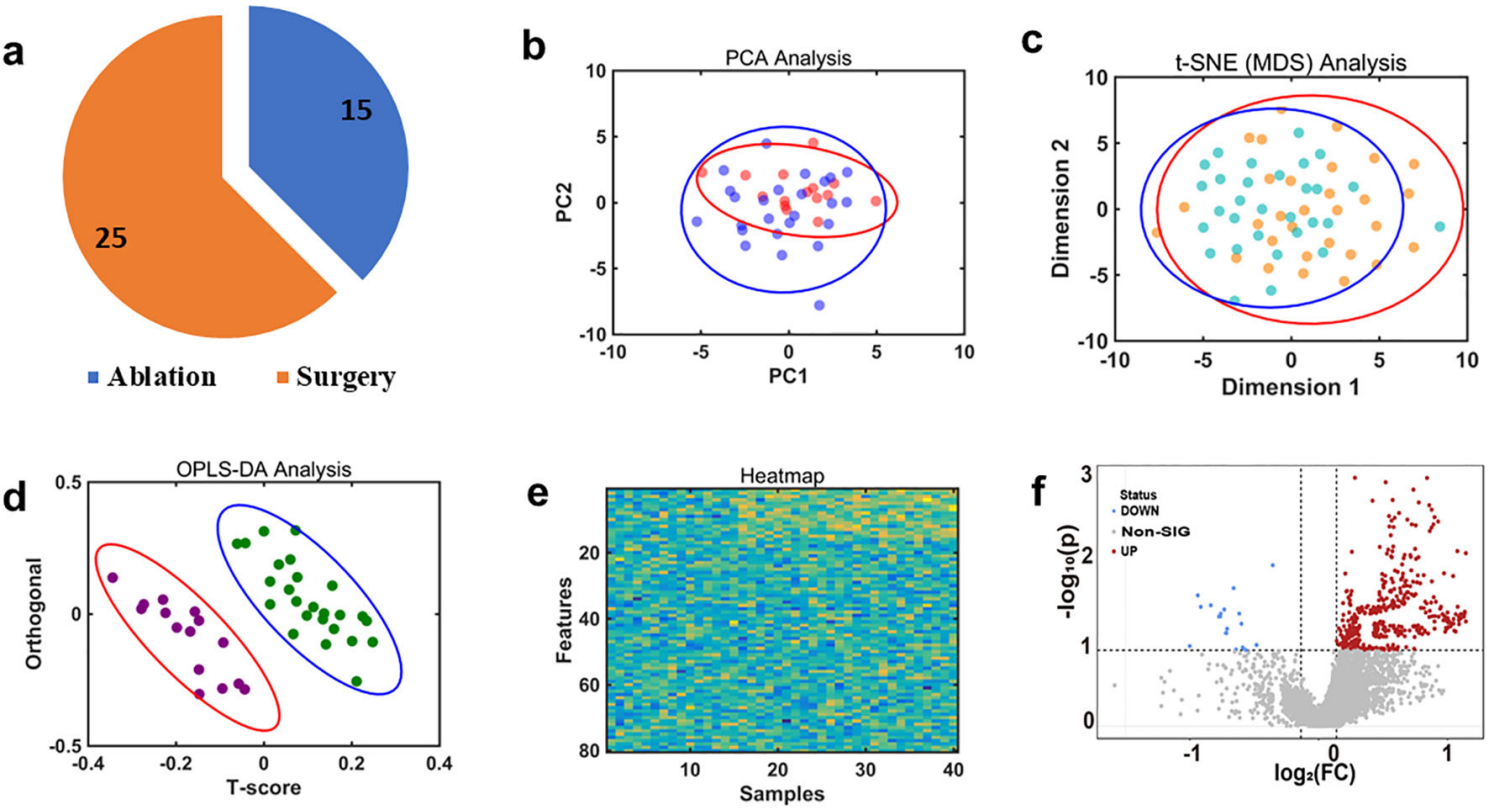
Metabolomic profiling distinguishes lung adenocarcinoma patients after ablation versus surgery. **(A)** Graphical representation of the study cohort’s baseline characteristics (n=40), establishing comparability between the ablation (n=15) and surgery (n=25) groups. **(B)** Principal Component Analysis (PCA) score plot showing substantial overlap between the two groups, indicating that unsupervised analysis fails to separate them based on global metabolic profiles. **(C)** t-SNE analysis plot similarly failing to achieve clear separation in an unsupervised manner. **(D)** Orthogonal Partial Least Squares Discriminant Analysis (OPLS-DA) score plot demonstrating a clear and distinct separation between the ablation and surgery groups, highlighting the model’s ability to uncover latent metabolic differences. **(E)** Heatmap visualizing the relative expression levels of numerous metabolites across all samples, revealing distinct clustering and differential expression patterns between the two treatment groups. **(F)** Volcano plot displaying significantly up-regulated (red) and down-regulated (blue) metabolites in the ablation group compared to the surgery group (fold change > 1.5, P < 0.05), confirming widespread metabolic reprogramming induced by the different treatments.

To clarify the distinct metabolic characteristics of lung adenocarcinoma patients after ablation versus surgery, samples from 15 patients in the ablation group and 25 in the surgery group were analyzed. Baseline information for the two groups was compiled and analyzed, as shown in **Figure 2A**, laying the foundation for subsequent metabolomic analysis. Furthermore, t-test analysis of patient age and other information showed no significant difference (p > 0.05, S1). The results of multidimensional statistical analysis showed that principal component analysis (PCA, **Figure 2B**) and t-SNE analysis (**Figure 2C**) failed to clearly distinguish between the two groups, with significant overlap in metabolic characteristics, indicating that unsupervised analysis alone was insufficient to identify differences.

However, further analysis using a supervised OPLS-DA model effectively separated the two types of samples clearly (**Figure 2D**), demonstrating good discrimination between groups and highlighting the model’s advantage in uncovering hidden metabolic differences. Simultaneously, heatmap analysis (**Figure 2E**) visually displayed a large number of differentially expressed metabolites between the two groups, with distinct stratification in expression levels. Volcano plot analysis (**Figure 2F**) further confirmed the presence of numerous significantly up-regulated and down-regulated metabolites between the groups, strongly suggesting that ablation and surgery, as two different treatment modalities, exert significant and distinct effects on the metabolic profiles of lung adenocarcinoma patients.

### 3.3 Comparison of diagnostic efficacy: metabolomics vs. traditional methods

**Figure 3:**
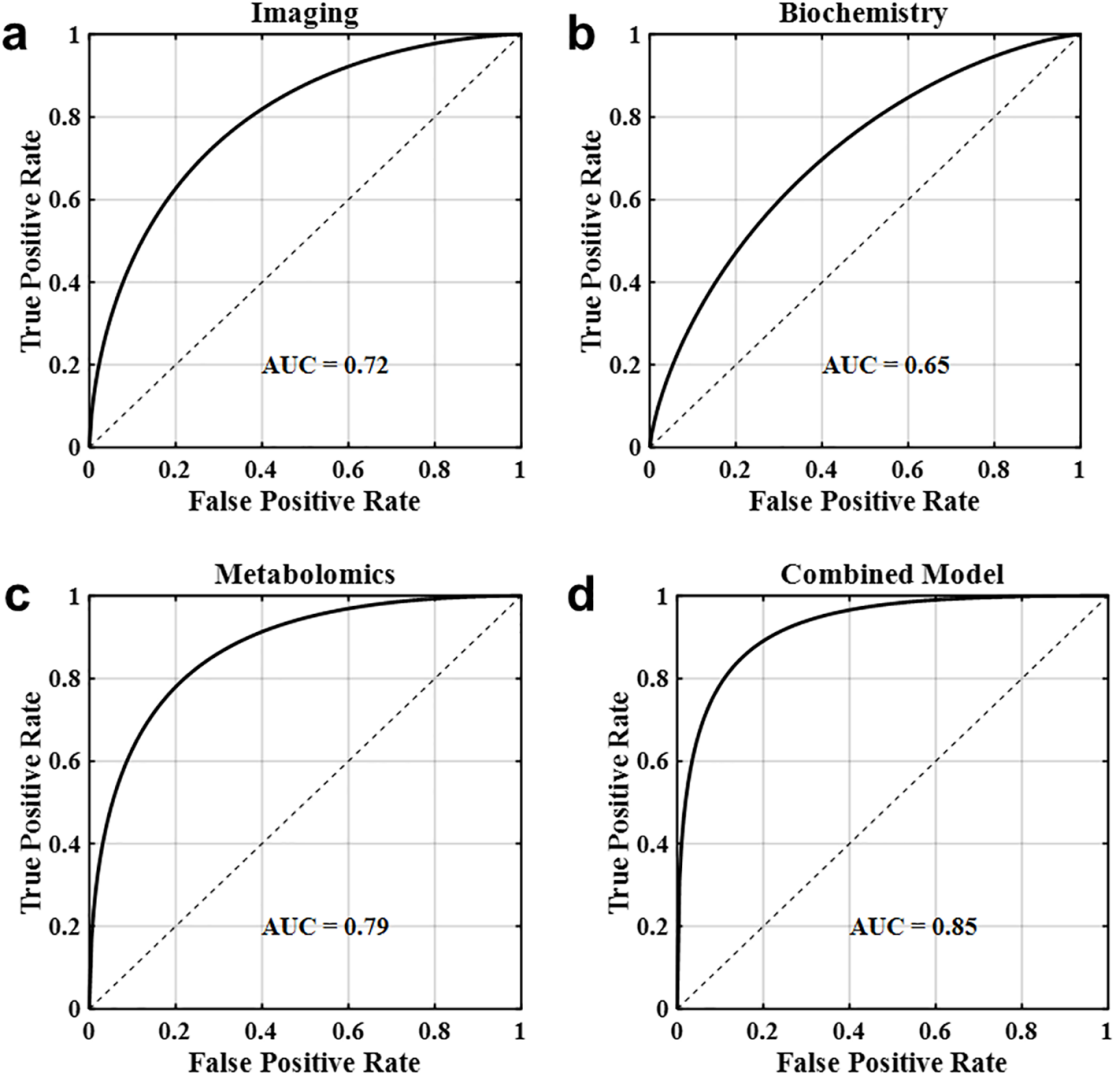
Diagnostic performance of different methods for discriminating ablation and surgery groups. Receiver operating characteristic (ROC) curve analysis comparing the area under the curve (AUC) for: **(A)** Traditional imaging methods (AUC = 0.75). **(B)** Traditional biochemical tests (AUC = 0.65). **(C)** Metabolomics-based model (AUC = 0.79). **(D)** Combined model integrating imaging, biochemical, and metabolomics data, showing a significantly improved AUC of 0.85, indicating superior discriminatory power for differentiating the two treatment modalities.

Building on the identification of differential metabolites, this study further compared the discriminatory power of metabolomics analysis horizontally with traditional imaging and biochemical testing methods. The results showed that the traditional imaging method had an AUC of 0.75 for distinguishing between the two groups (**Figure 3A**), while the traditional biochemical testing method had an even lower discriminatory AUC of only 0.65 (**Figure 3B**), indicating relatively low diagnostic efficacy. In contrast, the metabolomics method employed in this study demonstrated superior discriminatory ability, achieving an AUC of 0.79 (**Figure 3C**), with advantages in diagnostic sensitivity and specificity.

To further enhance the diagnostic efficacy for sample classification, this study combined imaging, biochemical testing, and metabolomics into a single model, integrating multi-dimensional detection information for comprehensive analysis. The results showed that the combined model’s AUC was significantly improved to 0.85 (**Figure 3D**), representing a substantial increase in discrimination accuracy. This finding strongly indicates that metabolomics can effectively assist in the differential evaluation of ablation and surgical treatment strategies, and its combination with traditional detection methods can further optimize the precision of clinical decision-making for lung adenocarcinoma.

### 3.4 Comparison of clinical indicators and advantages of ablation vs. surgery

To ensure the scientific validity of comparing clinical outcomes between the two treatment modalities, a balance test of patient baseline data was first conducted. Analysis of demographic characteristics showed no statistically significant difference in age between the ablation group (n = 15) and the surgery group (n = 25) (P > 0.05), confirming good comparability between the two cohorts at baseline and effectively excluding age as a potential confounding factor in the subsequent evaluation of clinical benefits.

**Figure 4:**
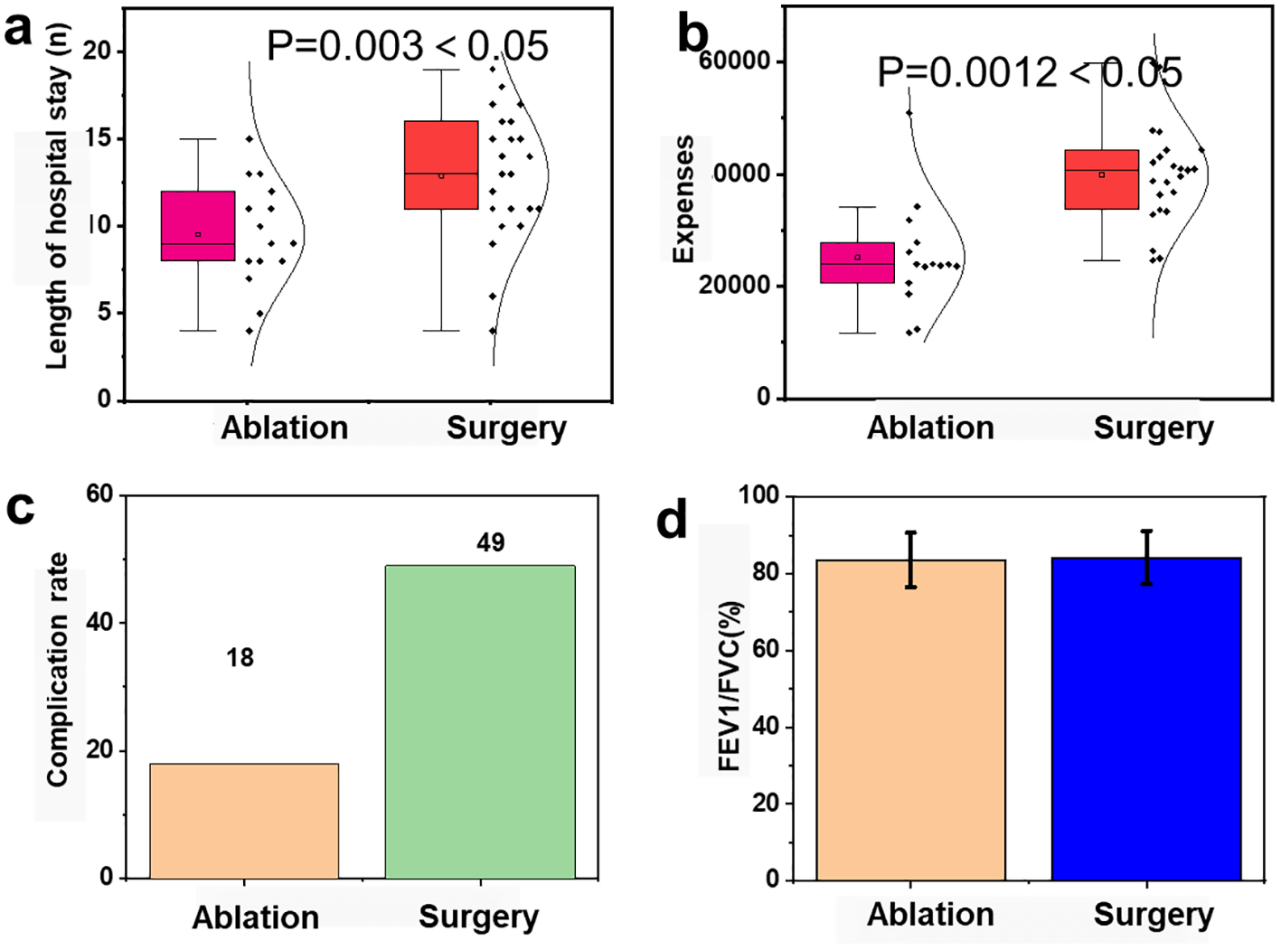
Comparison of clinical outcomes between ablation and surgery groups. Bar charts comparing key clinical indicators: **(A)** Hospital stay (days): Significantly shorter in the ablation group (P = 0.003). **(B)** Hospitalization costs (relative units): Significantly lower in the ablation group. **(C)** Complication rates (%): Significantly lower in the ablation group. **(D)** Pulmonary function (FEV1/FVC ratio): No significant difference between the two groups (P > 0.05), indicating equivalent preservation of lung function. Data are presented as mean ± SD. *P < 0.05, **P < 0.01 vs. Surgery group.

Systematic comparison of key clinical indicators revealed substantial advantages associated with ablation therapy. As shown in **Figure 4A**, the mean hospital stay was significantly shorter in the ablation group (9.2 ± 3.1 days) compared to the surgery group (12.7 ± 3.8 days), with the difference reaching statistical significance (P = 0.003). This reduction in hospitalization duration not only reflects the minimally invasive nature of the ablation procedure but also contributes to improved patient recovery and reduced bed occupancy rates. Regarding economic burden, the average hospitalization cost for patients undergoing ablation (23,529 ± 9,093 yuan) was markedly lower than that for surgical patients (40,112 ± 9,204 yuan) (**Figure 4B**), representing a substantial reduction in medical expenses that holds particular significance for health economics and patient affordability. Furthermore, analysis of postoperative safety profiles demonstrated that the ablation group experienced considerably fewer complication events (18 events) compared to the surgery group (49 events) (**Figure 4C**), despite the disparity in sample size. This lower complication rate underscores the superior safety profile of ablation and its advantage in preserving patient quality of life during the postoperative period.

Assessment of therapeutic efficacy focused on pulmonary function preservation, a critical outcome measure for lung cancer interventions. Evaluation of the core functional indicator FEV1/FVC revealed no significant difference between the two groups (ablation: 83.6 ± 7.1%; surgery: 84.2 ± 6.9%; P > 0.05) (**Figure 4D**), indicating that ablation achieves a level of pulmonary function preservation comparable to conventional surgery. This finding is particularly noteworthy as it demonstrates that the reduced invasiveness of ablation does not compromise its therapeutic effectiveness in maintaining respiratory function, thereby challenging the traditional paradigm that associates greater trauma with superior oncological outcomes.

## 4 Discussion

### 4.1 Application value of the mass spectrometry matrix in metabolomics analysis

Mass spectrometry is a core tool in metabolomics research, and the performance of the matrix directly determines the sensitivity, accuracy, and anti-interference ability of metabolite detection, making it a key prerequisite for ensuring reliable metabolomics results. The mass spectrometry matrix selected in this study produced clear and significant adduct peaks for two classic metabolites, phenylalanine and glucose, and maintained good linearity within reasonable concentration range. It meets both qualitative and quantitative detection requirements and is suitable for routine metabolite detection scenarios in lung adenocarcinoma.

Clinical samples have complex compositions, where impurities like salt ions and proteins can easily interfere with mass spectrometry signals. The validation results of this study confirmed that this matrix possesses excellent tolerance to salt and protein interference, effectively avoiding signal disruption from complex sample components and ensuring the stability and accuracy of detection results. This finding provides solid technical support for the subsequent metabolomic analysis of samples from lung adenocarcinoma patients and confirms the central role of a high-quality matrix in tumor metabolomics research, offering a reference for matrix selection in similar studies.

### 4.2 Metabolic differences and clinical significance of ablation vs. surgery for lung adenocarcinoma

Through multi-dimensional metabolomic analysis, this study found that ablation and surgery induce significantly different metabolic profiles in lung adenocarcinoma patients. Although unsupervised analyses like PCA and t-SNE failed to clearly separate the groups, the supervised OPLS-DA model successfully achieved precise sample discrimination. Combined with heatmaps and volcano plots identifying numerous differential metabolites, these results suggest that the two treatment modalities regulate the body’s metabolism through different mechanisms to exert their anti-tumor effects. Surgery primarily involves lesion resection and is a traumatic procedure with a significant impact on metabolic stress. In contrast, thermal ablation inactivates tumor cells through local thermal effects, causes less trauma, and its interference pattern with the body’s metabolism is more moderate. This is likely the core reason for the observed metabolic differences between the two groups.

These metabolic differences not only provide a molecular basis for distinguishing the two treatment modalities but also offer a new direction for exploring the anti-tumor mechanisms of different procedures and optimizing treatment plans. By investigating the biological functions of the differential metabolites, the specific targets of ablation and surgery can be further clarified, providing theoretical support for individualized treatment of lung adenocarcinoma and laying a foundation for evaluating therapeutic effects from a metabolic perspective.

### 4.3 Diagnostic value of metabolomics combined with traditional tests and clinical advantages of ablation

Traditional imaging and biochemical tests are routine methods for lung adenocarcinoma diagnosis and treatment, but they suffer from limitations such as insufficient diagnostic sensitivity and low discriminatory efficacy. In this study, their AUC values were only 0.75 and 0.65, respectively, falling short of the requirements for precision medicine. Metabolomics can capture subtle changes related to the disease and treatment from the body’s metabolic state, achieving a diagnostic AUC of 0.79 and demonstrating superior discriminatory ability. Combining the three methods into a model yielded a high AUC of 0.85, achieving complementary information from multiple dimensions and significantly enhancing diagnostic accuracy. This confirms that metabolomics holds significant auxiliary value in the diagnosis and treatment of lung adenocarcinoma, effectively optimizing treatment strategy differentiation and efficacy evaluation.

The comparison of clinical indicators further solidifies the clinical advantages of ablation. With no age difference between the groups ensuring objective comparison, the ablation group was significantly superior to the surgery group in terms of hospital stay, cost, and complications, while maintaining comparable pulmonary function. This challenges the traditional notion of a direct correlation between therapeutic trauma and efficacy. For elderly, frail patients with early-stage lung adenocarcinoma who cannot tolerate surgery, ablation can serve as a safe and effective alternative, offering both minimal invasiveness and reliable efficacy, aligning with the clinical trend towards minimally invasive and precise treatment. This study also fills a gap in controlled research comparing thermal ablation and surgery, providing empirical support for optimizing clinical treatment choices.

## 5 Conclusion

This study validated that the selected MALDI-MS matrix possesses excellent detection sensitivity and anti-interference capability, suitable for metabolomic analysis in lung adenocarcinoma. Metabolomic profiling revealed distinct metabolic signatures between ablation-treated and surgically treated patients. Integration of metabolomics with traditional imaging and biochemical tests significantly improved diagnostic efficacy for treatment differentiation (AUC = 0.85). Clinical assessment demonstrated that ablation therapy offers significant advantages in shortening hospital stays, reducing medical costs, and lowering complication rates, while preserving pulmonary function comparable to surgery. These findings position metabolomics as a valuable auxiliary tool for treatment evaluation and support thermal ablation as a promising minimally invasive alternative for early-stage lung adenocarcinoma. Nevertheless, this study has limitations including a relatively small sample size and the need for further validation of the underlying metabolic mechanisms. Further studies with expanded cohorts and extended follow-up are warranted to validate these findings and explore the functional roles of differential metabolites.

## Data Availability

The original contributions presented in the study are included in the article/**Supplementary Material**. Further inquiries can be directed to the corresponding author.
