# Supplementary material for: Metabolomics-assisted evaluation of differences and clinical value between ablation and surgical treatment for lung adenocarcinoma
Source: Front Oncol. 2026 Jul 16;16:1836590. doi: 10.3389/fonc.2026.1836590 (PMC13422148; doi:10.3389/fonc.2026.1836590)
Supplement: Supplementary file 1 [file DataSheet1.docx]

Metabolomics-Assisted Evaluation of Differences and Clinical Value between Ablation and Surgical Treatment for Lung Adenocarcinoma

Youli Wen^#^, Yang Zeng^#^, Wenqiang Li, Xinyu Deng, Wu Qunhua, Prof. Zhiping Deng^*^

Department of Respiratory and Critical Care Medicine, Zigong First People's Hospital, Zigong, China.

***Correspondence:** Prof. Zhiping Deng, E-mail address: [dengzp1016@163.com](mailto:dengzp1016@163.com)

^#^Youli Wen and Yang Zeng contributed equally.

Keywords: metabolomics, MALDI-MS, surgery, lung adenocarcinoma.

.


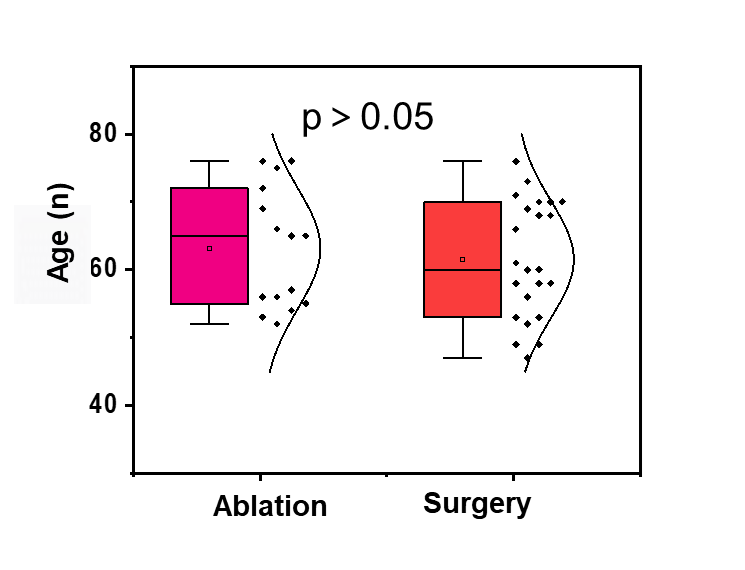


**S1.** The t-test analysis of age between the ablation group and the surgery group demonstrated a result of > 0.05, indicating no significant difference.
